# The inhibiting effect of alpha-based TARE on embolized vessels and neovascularization

**DOI:** 10.3389/fbioe.2022.1021499

**Published:** 2022-10-07

**Authors:** Qianqian Tong, Rou Li, Ruizhi Wang, Changjing Zuo, Danni Li, Guorong Jia, Ye Peng, Xiaohong Li, Jian Yang, Shuai Xue, Qingyun Bai, Xiao Li

**Affiliations:** ^1^ School of Chemistry and Bioengineering, Yichun University, Yichun, Jiangxi, China; ^2^ Department of Nuclear Medicine, Changhai Hospital, Naval Medical University, Shanghai, China; ^3^ Department of Radiology, Huadong Hospital, Fudan University, Shanghai, China; ^4^ Institute of Applied Physics, Chinese Academy of Sciences, Shanghai, China

**Keywords:** hepatocellular carcinoma, transarterial radioembolization, α radionuclides, Ra-223, neovascularization, silk fibroin, necrosis

## Abstract

Transarterial embolization (TAE) is a personalized technology that offers precise delivery of chemotherapeutic drugs or selective internal radiation therapy for hepatocellular carcinoma (HCC). Beta-emitting radionuclide embolisms for TAE (β-based TARE) are commonly used in the clinic *via* inducing biochemical lethality on tumor cells, while alpha-emitting radionuclides-based embolisms for TAE (α-based TARE) are still under study. The feeding artery plays a key role in tumor growth, metastasis, and recurrence. In this research, the auricular central arteries (ACAs) of rabbits were embolized with silk fibroin-based microspheres (SFMs) or SFMs integrated with α (Ra-223) or β (I-131) radionuclides to investigate the influence on vessels. TARE-induced tissue necrosis and the following neovascularization were measured by pathological analysis and ^68^Ga-DOTA-RGD PET/CT. The results showed that, compared to I-131, Ra-223 enhanced the growth inhibition of human hepatoma cells Huh-7 and induced more DNA double-strand breaks in vascular smooth muscle cells. Unlike β-based TARE, which mainly led to extensive necrosis of surrounding tissues, α-based TARE induced irreversible necrosis of a limited area adjacent to the embolized vessels. RGD PET revealed the inhibition on neovascularization in α-based TARE (SUV_max_ = 0.053 ± 0.004) when compared with normal group (SUV_max_ = 0.099 ± 0.036), the SFMs-lipiodol group (SUV_max_ = 0.240 ± 0.040), and β-based TARE (SUV_max_ = 0.141 ± 0.026), owing to the avoidance of the embolism-induced neovascularization. In conclusion, α-based TARE provided a promising strategy for HCC treatments *via* destroying the embolized vessels and inhibiting neovascularization.

## 1 Introduction

Liver cancer is a major global health challenge, and hepatocellular carcinoma (HCC) is the most common (90%) type of primary liver cancer (Llovet et al., 2021). As a treatment with precise delivery of radioactive nuclides to the tumorous feeding artery, transarterial radioembolization (TARE) delivers therapeutic isotopes targeting the tumor in patients with unresectable hepatic malignancy (Habib et al., 2015; Bruix et al., 2016). Recently, with the development of nuclear technology and innovation of theranostic equipment, a number of radioactive microspheres and particles for TARE have become available.

So far, three types of beta-emitting radionuclide embolisms for TARE (β-based TARE) have been applied in the clinic. These β-emitting radionuclides generate β-rays with linear energy (0.1–2.2 MeV) to produce reactive oxygen species (ROS), leading to the mitochondria-related cellular apoptosis or damage to the single-strand DNA of cells (Aghevlian et al., 2017; King et al., 2021). The range of β-emitting is over 1 mm, an effective range to cover most solid tumors when beads are embolized in the peripheral artery. In the clinic, yttrium-90 (Y-90) microspheres, including TheraSpheres and SIR-Spheres, have already been applied in unresectable HCC or unresectable liver metastases of colorectal cancer (Edeline et al., 2015). Recently, a type of holmium-166 (Ho-166) microspheres named QuiremSpheres, which were developed with biodegradable poly-l-lactic acid-based microspheres containing Ho-166, have been approved to treat unresectable liver cancer (Reinders et al., 2019). Another extensively used β-emitting radionuclide is iodine-131 (I-131), which has several advantages, including economic feasibility and easy securement (Fujiwara, 2016). In detail, I-131 has a half-life of 8.06 d (t_1/2_ = 8.06 d) and emits β particles of maximum energy of 0.6 MeV with a 2.3 mm maximum range in tissue (Mies et al., 1981), which could penetrate the embolized arterial walls and reach the boundary of tumorous tissues (Li et al., 2021). Hence, β-emitting radionuclides like Y-90, Ho-166, and I-131 are commonly developed or being developed for β-based TARE.

Different from β-emitting radionuclides, α-emitting radionuclides are among the most radiotoxic of all radionuclides. Although the radionuclides (such as iodine-125) with Auger electrons are of nuclear toxicity, the requirements for entrance into cells and approaching the cell nucleus limit the opportunity to translate these properties into clinical therapies. Usually, α nuclides permit a short range of radiation of less than 100 μm (Kratochwil et al., 2014), but the linear energy is 100–1,000 times greater than conventional β-emitting radionuclides (Sgouros, 2008). Along the emitting track *in vivo*, α-particles could emit thousands of ion pairs with radiation from enormously higher linear energy (4–9 MeV) to deposit on and break the double-strand DNA (Heskamp et al., 2017). Therefore, α-emitting radionuclides irreversibly accelerate the death of the exposed cells. For example, radium-223 (Ra-223) has a half-life of 11.43 d (t_1/2_ = 11.43 d), and four emissions of α-particles are generated during each decay. These particles predominately break double-strand DNA, which accounts for the irreversible cell damage (Pandit-Taskar et al., 2014; Roobol et al., 2020). In addition to cellular death, embolization-induced neovascularization is the first concern in controlling recurrence and metastasis. However, in the application of alpha-emitting radionuclide-based embolisms for transarterial embolization (α-based TARE), the systemic influence of the α-emitter in the whole procedure of embolization, especially its effect on the embolized vessels and the following vascular remodeling, was not understood.

**FIGURE 1:**
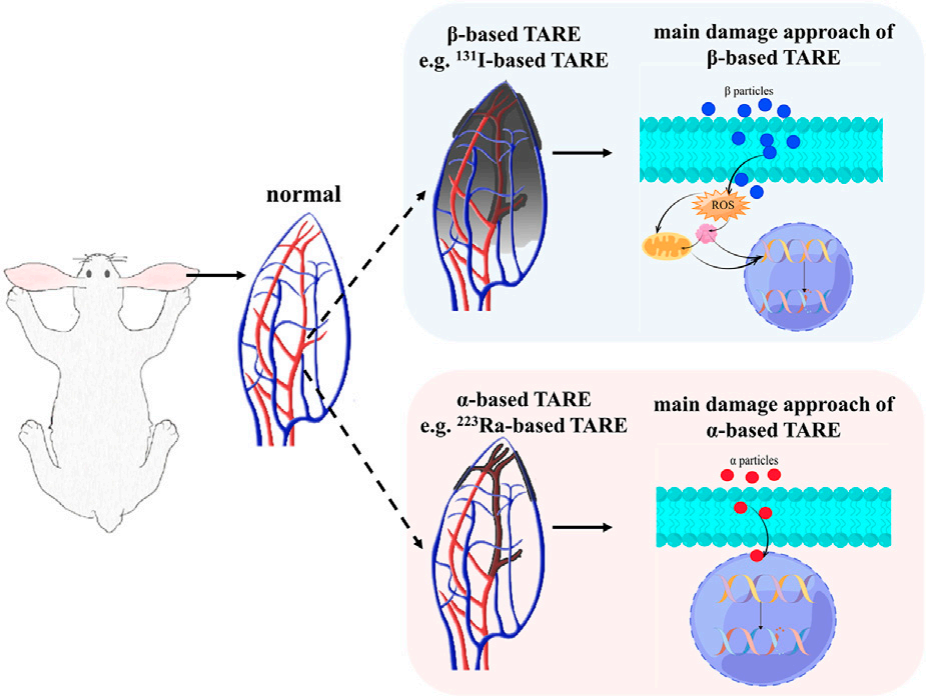
The schematic radiation process and the main damage approaches of β-based TARE and α-based TARE. The shaded area represents the radiation coverage area.

Until now, fewer α-emitting radionuclides have been used in TARE. This present study aims to determine the principles of α-based TARE, especially the effect of radiation on the embolized artery and surrounding tissues. Ra-223 was chosen as the representative radionuclide in exploring α-based TARE, and I-131 served as a comparison with definite therapeutic effects (Figure 1). Silk fibroin microspheres (SFMs) offer the characteristics of relatively good dispersion stability, biodegradability, biocompatibility, and low cytotoxicity (Sun et al., 2018; He et al., 2020), and the feasibility of radionuclide-labeled SFMs for TARE to treat HCC has been proven (Wu et al., 2022). In this study, we compared the ability of I-131 and Ra-223 to induce apoptosis at the cellular level, the inhibition effects on tumor cell growth, and the induction of γ-H2AX in vascular smooth muscle cells (VSMCs). SFMs combined with Ra-223 or I-131 were injected into the auricular central artery (ACA) of rabbits, where the radionuclides served as the unique variable. Single photon planar imaging was used to quantify the efficiency of embolization, and the subsequent damage to the appearance and pathological section of the ear were recorded. Functional imaging of neovascularization was performed using positron emission tomography/computed tomography (PET/CT) imaging with gallium-68-labeled 1,4,7,10-tetraazacyclododecane-1,4,7,10-tetraacetic acid conjugated with arginine-glycine-aspartate (^68^Ga-DOTA-RGD) post TARE, and the specific findings were verified with pathological analysis.

## 2 Materials and methods

### 2.1 Materials and animals

Na^131^I was purchased from Shanghai XinKe Pharmaceutical Co., Ltd. ^223^RaCl_2_ was purchased from the Institute for Energy Technology. The operation related to the above agents was performed in a hot cell. Ethiodized poppyseed oil injection was purchased from Jiangsu HengRui Medicine Co., Ltd. Silk fibroin microspheres (about 150.61 ± 10.39 μm in diameter, controlled by steel screens) were purchased from Suzhou Meilun Biotechnology Co., Ltd. The TdT-mediated dUTP Nick-End Labeling (TUNEL) kit was purchased from KeyGEN Biotechnology Co., Ltd. The annexin V-fluorescein isothiocyanate (FITC)/propidium iodide (PI) apoptosis kit was purchased from BD Bioscience. A cell counting kit-8 (CCK-8) was purchased from the Beyotime Institute of Biotechnology. A γ-H2AX ELISA kit was purchased from Jiangsu Meimian Industrial Co., Ltd.

A human hepatocyte carcinoma cell line (Huh-7) was purchased from the Cell Bank of the Type Culture Collection of the Chinese Academy of Sciences, Shanghai Institute of Cell Biology, Chinese Academy of Sciences. The New Zealand white rabbits (male, weighing 2.5 ± 0.5 kg) were purchased from Ailingfei Biotechnology Co., Ltd., and kept under a specific pathogen-free condition at the laboratory animal center. This research was approved and guided by the Ethics Committee of Naval Medical University (Approval No. 1207050662).

### 2.2 Cell culture

Primary mouse VSMCs were isolated from C57BL/6 mice as previously described (Chamley-Campbell et al., 1979). VSMCs were grown to confluence in DMEM medium supplemented with 10% FBS and 1% penicillin-streptomycin. Huh-7 cells were cultured in DMEM containing 10% FBS at 37°C in 5% CO_2_.

### 2.3 Flow cytometry

VSMCs and Huh-7 cells at a density of 10^5^ cells per sample were incubated with I-131 (1 mCi) or Ra-223 (1 μCi) for 24 h at 37°C, respectively. Post incubation, the cells were washed twice with cold PBS and then stained using the annexin V-FITC/PI apoptosis kit, followed by flow cytometric analysis. The percentage of apoptosis was corrected for background levels found in the corresponding untreated controls. The percentage of cells undergoing apoptosis was defined as the sum of early apoptosis (annexin V-positive and PI-negative) and late apoptosis (annexin V-positive and PI-positive) cells. Necrotic cells of VSMCs and Huh-7 cells were defined as the percentage of annexin V-negative and PI-positive cells. Three independent experiments were performed, and the values of apoptotic percentages were presented as the mean ± SD.

### 2.4 Cell viability and γ-H2AX activation assays

The cell viability assay was performed on tumor cells using CCK-8. Huh-7 cells were seeded into 96-well culture plates at a density of 1 × 10^4^ cells per well in 100 μl culture medium and cultured overnight at 37°C. Three duplicate wells were set up for each group. The cells were then exposed to I-131 (100 μCi) or Ra-223 (0.1 μCi) for 24 h, then 10 μl CCK-8 solution was added to each well. After incubating for 4 h at 37°C in 5% CO_2_, absorbance at 450 nm was measured using a microplate reader.

For the γ-H2AX assay, VSMCs were seeded into 96-well culture plates at a density of 1 × 10^4^ cells per well and incubated overnight at 37°C. VSMCs were treated with I-131 (100 μCi) or Ra-223 (0.1 μCi) for 24 h. Subsequently, the treated VSMCs were fixed, permeabilized, and stained with anti-γ-H2AX following the γ-H2AX ELISA kit protocol. The induced concentration of γ-H2AX was quantified on the basis of absorbance at 450 nm.

### 2.5 Preparation of embolism and radiopharmaceuticals

To make SFMs-lipiodol TAE, 50 μg SFMs were added into 1 ml 0.9% saline to obtain an SFMs suspension. Afterward, 2 ml ethiodized poppyseed oil injection was added to the SFMs suspension. The SFMs-lipiodol suspension was further aspirated and blown with a pipette and then sonicated for 1 minute to ensure the homogeneous dispersion of microspheres.

To make ^131^I-SFMs-lipiodol TARE, 1 ml Na^131^I (6.6 MBq) was mixed with 50 μg SFMs and vibrated for 10 min to increase physical adsorption between I-131 and SFMs. The adsorption rate was evaluated with thin-layer chromatography (TLC) with a radioactive detector. Glass microfiber chromatography paper impregnated with silica gel was used as the stationary phase, and saline was used as the mobile phase. The free I-131 was removed by centrifugation if the adsorption rate was less than 90%. Then, 2 ml ethiodized poppyseed oil was added to ^131^I-SFMs to obtain the suspension of ^131^I-SFMs-lipiodol. The β-based TARE of each ACA used 1.1 MBq/0.5 ml ^131^I-SFMs-lipiodol.

To make ^223^Ra-SFMs-lipiodol TARE, 1 ml Ra-223 (6.6 kBq) was mixed with 50 μg SFMs and vibrated for 10 min to increase the physical adsorption of Ra-223 to SFMs. The quality control and purification were the same as I-131. After that, 2 ml ethiodized poppyseed oil was added to the mixture and mixed as described above. The ^223^Ra-SFMs-lipiodol was suspended, and 1.1 kBq/0.5 ml was used for the α-based TARE of each ACA.

^68^Ga^3+^ was acquired in the form of ^68^GaCl_3_ from a^68^Ge/^68^Ga generator by elution with 0.1 M HCl and then labeled to the integrin-targeted DOTA-RGD in house following the protocols. Quality control of ^68^Ga-DOTA-RGD was performed to guarantee the radiochemical purity >90%. The injected dosage of each rabbit was set as 7.4 MBq/kg body weight.

### 2.6 Radioembolization of auricular central arteries

In consideration of the visibility of ACAs, the strategy of ACA embolization was chosen to verify α-TARE (Li et al., 2021). ACA embolization of 12 rabbits was performed to evaluate the differential radiation damage to the artery and surrounding tissues. Before embolization, the ears of New Zealand rabbits were shaved and cleaned. Afterward, the rabbits (*n* = 3 for each group) were divided into four groups: a normal group, an SFMs-lipiodol group, a ^131^I-SFMs-lipiodol group, and a ^223^Ra-SFMs-lipiodol group. The rabbits were anesthetized under 3% (v/v) isoflurane. The suspension was emulsified before injection to prevent delamination. Subsequently, a 500 µl suspension of the embolism was slowly injected along the flow of blood into the ACA. The injection site was pressed for 5 min to control the bleeding and backflow. By virtue of α-particles depositing energy 100 to 1,000 times higher than β-particles, the activity of Ra-223 was adjusted to 1.1 kBq per ear, while the activity of I-131 was set as 1.1 MBq. Rabbits were excluded from the study if the recanalization of the ACA occurred after the backflow of blood in the arteries. The changes in the ear appearance were photographed at 1, 3, 5 and 7 days after the embolization.

### 2.7 Single photon planer imaging on embolization and radiation inspection

Single photon planar imaging was performed to record the distribution of SFMs-lipiodol. A SPECT/CT (Symbia T16, Siemens) scanner was utilized, and scans were performed immediately after the TARE and at 48 h post-injection to detect the distribution of ^131^I-SFMs-lipiodol *in vivo.* I-131 possesses an energetic spectrum of gamma radiation (Eγ = 0.364 MeV), and the ^131^I-SFMs-lipiodol group was chosen as the representative one.

During scan acquisition, rabbits were anesthetized under 3% (v/v) isoflurane. In addition, ears were stretched away from the body to avoid signal interference from other organs. The SPECT/CT scanner was equipped with a high-resolution collimator, and the acquisition of planar pictures was performed in 5 min with matrix = 256 × 256 and zoom = 1.

Radiation from ^131^I-SFMs-lipiodol was measured by a Radiation Alert^®^ inspector to quantify the embolization efficiency. The radiation from the whole body and the body except for the embolized ear was measured at 0, 36, 72, and 120 h post embolization. Embolized dosage (ED) was used in this study to represent the results, which was similar to “ID” in SPECT/CT. The time-dependent residuals in the embolized ear were calculated, and the ratio of the embolization in the ear to the whole body and the residual percentage of embolization in the ear are presented as line charts.

### 2.8 ^68^Ga-DOTA-RGD PET/CT imaging

^68^Ga-labeled arginine-glycine-aspartate peptides were regarded as PET tracers for angiogenesis imaging because RGD is specific toward the integrin α_v_β_3_ that is excessively expressed during neovascularization (Parihar et al., 2020). To compare the state of the neovascularization of ears after embolization in each group, a ^68^Ga-DOTA-RGD PET/CT scan was performed at 4 days post-embolization. About 7.4 MBq/kg of ^68^Ga-DOTA-RGD were administrated through the ear vein, and PET/CT images were acquired 45 min later. The spatial resolution of the PET/CT scanner (Biograph 64; Siemens) was 1 mm with CT (120 kV, 35 mA). In addition, the reconstruction resolution of CT was 1 mm by the postprocessing workstation TureD system. The tracer uptake of ^68^Ga-DOTA-RGD was presented as the maximum standardized uptake value (SUV_max_).

### 2.9 Histopathological examination

After 7 days post-embolization, all rabbits were sacrificed with excessive anesthesia. All ears were harvested, and the selected tissues mainly included the auricular artery and surrounding tissues, which were fixed and preserved in 10% neutral buffered formalin for 48 h. Thereafter, the fixed tissues were dehydrated, transparentized, embedded in paraffin, and longitudinally cut into 4-μm thick slices. Slices were stained with hematoxylin-eosin (H&E) and TUNEL and then photographed under a microscope equipped with optical and fluorescent lenses.

### 2.10 Statistical analysis

The values of SUV_max_ were described as the mean ± standard deviation (SD). Comparisons were performed using an unpaired *t*-test. The analyses of histopathological examination were performed using ImageJ software.

## 3 Results

### 3.1 Cell damage by radiation

**FIGURE 2:**
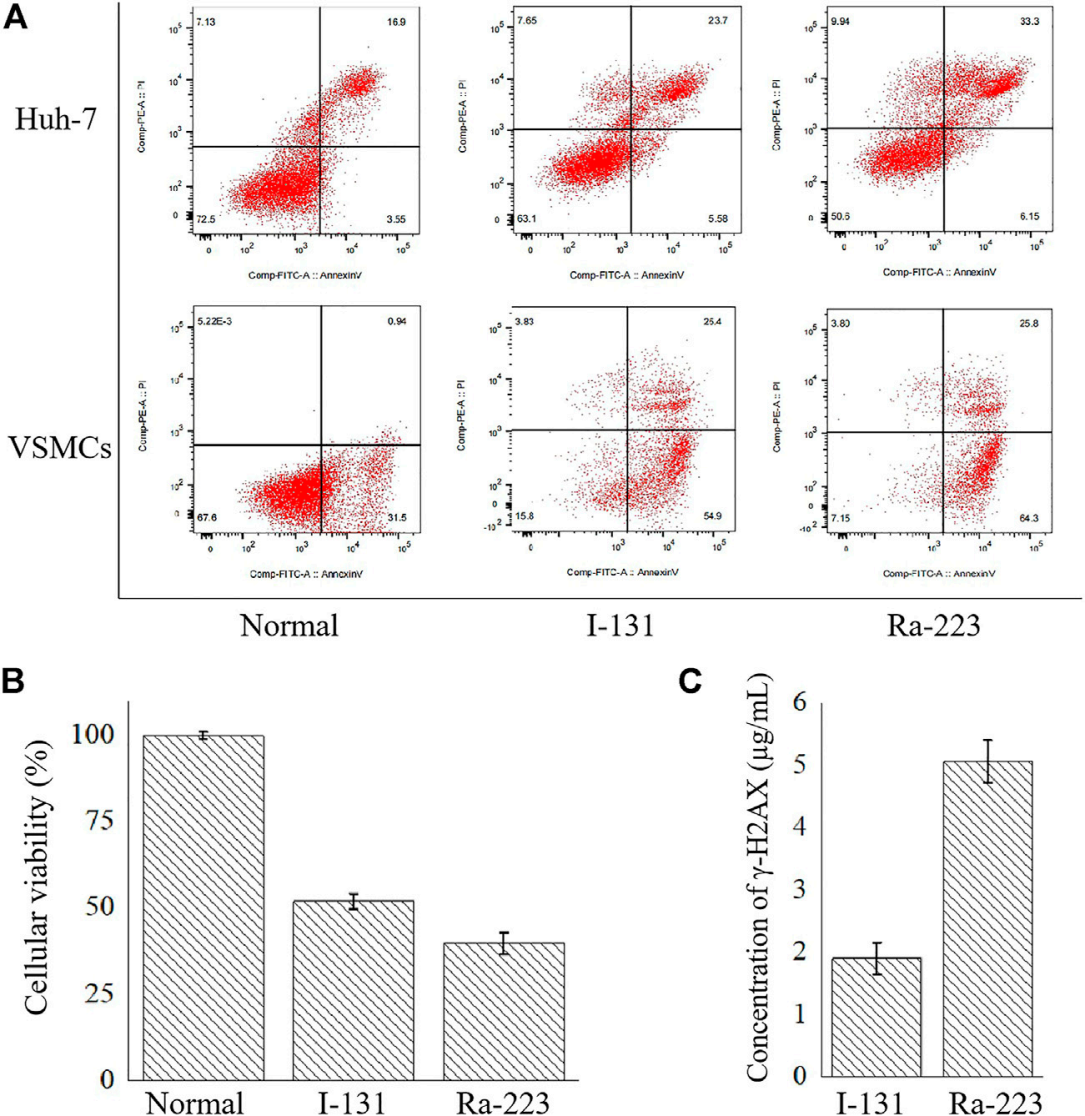
The comparison between I-131 and Ra-223 on the cellular level, including the apoptosis induction **(A)**, the inhibition of the cellular viability of Huh-7 cells **(B)**, and the induction of γ-H2AX on VSMCs **(C)**.

Apoptosis and necrosis were the main consequence of radiation. The effects of 24 h radiation of radionuclides on Huh-7 and VSMCs were examined using flow cytometry on apoptosis. As shown in Figure 2A, compared with the normal group, apoptotic percentages were elevated in Huh-7 cells and VSMCs after the treatment of I-131 and Ra-223. Compared with normal Huh-7 cells (20.20% ± 0.70%), the percentage of apoptotic Huh-7 cells increased to 29.33% ± 0.08% after treatment with I-131 and 39.93% ± 0.03% after treatment with Ra-223. Compared with normal VSMCs (31.15% ± 1.38%), the percentage of apoptotic VSMCs increased to 79.41% ± 1.41% after treatment with I-131 and 88.88% ± 0.29% after treatment with Ra-223. Although I-131 and Ra-223 both induced apoptosis, the effects of Ra-223 in Huh-7 cells at the late stage of apoptosis and in VSMCs at the early stage of apoptosis were more obvious than I-131. In addition, the percentage of necrotic Huh-7 cells increased to 7.81% ± 0.26% after treatment with I-131 and 9.87% ± 0.21% after treatment with Ra-223. The percentage of necrotic VSMCs increased to 4.22% ± 0.31% after treatment with I-131 and 4.13% ± 0.30% after treatment with Ra-223.

In order to determine the influences of treatment on tumorous cell viability, the cellular viability of Huh-7 cells co-incubated with I-131 or Ra-223 for 24 h was examined *via* the CCK-8. Compared to the control group, treatments with I-131 and Ra-223 signally inhibited Huh-7 cell viability (Figure 2B), and treatment with Ra-223 was more effective in inhibiting Huh-7 cell proliferation.

Compared to the DNA strand breakage caused by the I-131-induced γ-H2AX (1.89 ± 0.05 μg/ml), the treatment of Ra-223 further increased γ-H2AX expression levels (5.05 ± 0.05 μg/ml) (Figure 2C), creating a more thorough lethality.

### 3.2 Radioembolization of auricular central arteries

^131^I-SFMs and ^223^Ra-SFMs were successfully synthesized *via* the adsorption capacity of protein in silk fibrin. Although the physical adsorption was of relatively low stability, this approach was the most effective way of establishing the unbiased protocol to only evaluate the influence of nuclides, regardless of confounding factors, such as the chemical modification and labeling method. In addition, the ^68^Ga-DOTA-RGD synthesized in house met the quality control criteria of acid-base property (pH = 4), radiochemical purity (>95%), and solubility (clear and no undissolved substance). The specificity activity was adjusted to around 37 MBq/µg precursor.

**FIGURE 3:**
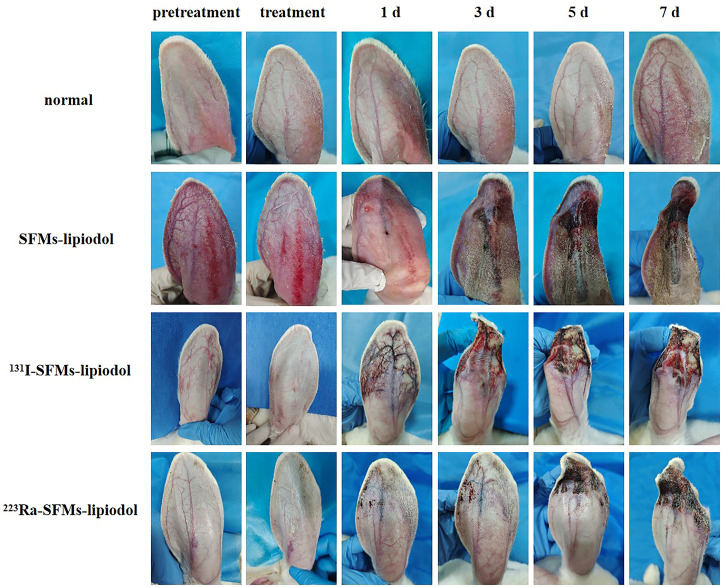
The appearance of pre-embolization rabbit ears, immediately post treatment with SFMs-lipiodol, ^131^I-SFMs-lipiodol and ^223^Ra-SFMs-lipiodol, and at 1, 3, 5, and 7 days post-embolization.

As time progressed, progressive changes in skin color and appearance of the rabbit ears were observed in the embolization groups. As shown in Figure 3, the ACAs, auricular veins, and terminal branches of vessels were immediately pale after injection, owing to the blockage of blood flow. After embolization for 1 day, the vascular color in the ACA changed to purple in the embolization groups (the SFMs-lipiodol group, the ^131^I-SFMs-lipiodol group, and the ^223^Ra-SFMs-lipiodol group). Distal vessels and tissues around the embolized arteries became maroon in the ^131^I-SFMs-lipiodol group. By comparison, the surrounding tissues remained normal during the super early period of embolization in the ^223^Ra-SFMs-lipiodol group. On the third day after embolization, the congestive edema of ears in the SFMs-lipiodol group emerged, and then necrosis occurred in a portion of the tissue. Extensive necrosis and shrinkage of ears were observed in the ^131^I-SFMs-lipiodol group. Comparably, no recanalization of the ACA occurred, and tissues around arteries only slightly turned purple in the ^223^Ra-SFMs-lipiodol group at 3 days post-embolization. Significant changes could be seen in the ^223^Ra-SFMs-lipiodol group from the fifth to the seventh day post-embolization. The distal tissue supplied by the embolized vessel became black, stiff, and twisted, while the adjacent and proximal tissues showed no damage. Conversely, some normal non-necrotic tissues were scattered throughout the extensive necrosis in the ^131^I-SFMs-lipiodol group at 7 days post-embolization. The progressive changes in ears indicated that ^223^Ra-SFMs-lipiodol TARE induced restricted and thorough damage, which resulted from the shorter radiation range and higher energy of α-particles than β-particles.

### 3.3 Distribution of SFMs-lipiodol in TARE

**FIGURE 4:**
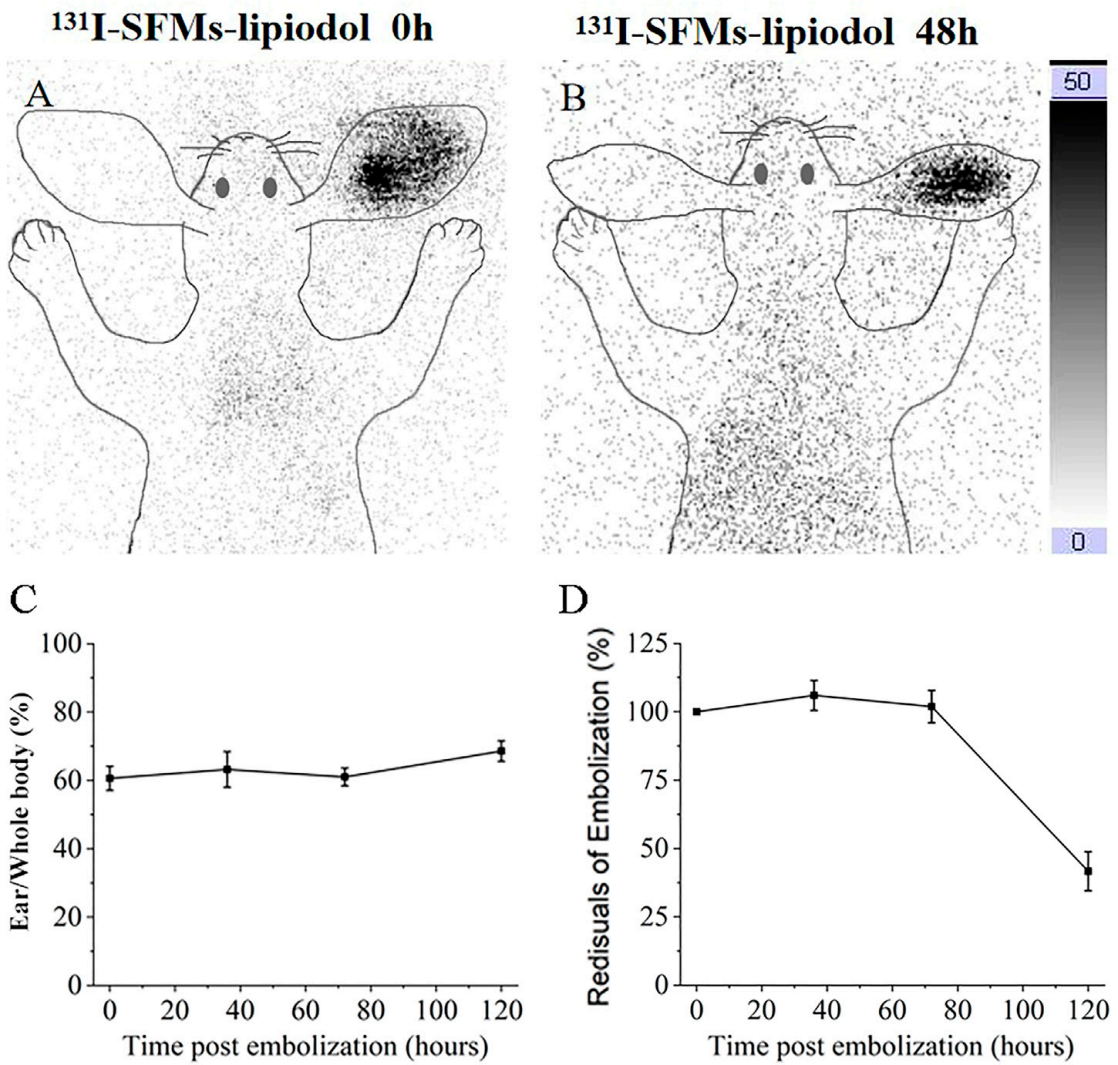
Planar images of rabbit at 0 h **(A)** and 48 h **(B)** after embolization with ^131^I-SFMs-lipiodol. Ear to whole body radioactivity ratios were measured at 0, 36, 72, and 120 h **(C)**. Residual radioactivity ratios of embolization (decay corrected) were measured at 0, 36, 72, and 120 h **(D)**.

Because there was no γ radiation in the normal and the SFMs-lipiodol groups and a low dose of γ radiation in the ^223^Ra-SFMs-lipiodol group, the ^131^I-SFMs-lipiodol group was examined at 0 and 48 h after embolization. Because the same embolism procedure was used in other groups, except for the radionuclides, planar imaging of the ^131^I-SFMs-lipiodol group revealed the distribution of embolism in all groups. Compared with the surrounding tissue, an embolism in the tip of the ear was distinguishable in the ^131^I-SFMs-lipiodol group after injection, as presented in Figure 4A. The anchored radioactivity in the ACAs increased to 60.21% embolized dosage (ED) after the primary distribution. At 48 h after embolization, the leaked ^131^I-SFMs was distributed *in vivo* without a specific target, as shown in Figure 4B. Meanwhile, 16.37% of the SFMs-lipiodol was excreted from the rabbit. Illustrated data visually demonstrated that ^131^I-SFMs-lipiodol deposited in the ACA and provided continuous radiation to the ACA and its surroundings. Images confirmed that SFMs-lipiodol could potentially be used as the base of TARE.

In terms of quantification, ear-to-whole-body radioactivity ratios were maintained at a high level (60%–70%) at 0, 36, 72, and 120 h (Figure 4C). Results confirmed ^131^I-SFMs-lipiodol demonstrated occlusion in the ACA and had a favorable *in vivo* distribution. The residual radioactivity ratio of decay-corrected embolization decreased between 72 and 120 h (Figure 4D), consistent with the timeline of extensive necrosis in ^131^I-SFMs-lipiodol group shown in Figure 3.

### 3.4 The inhibition of neovascularization

**FIGURE 5:**
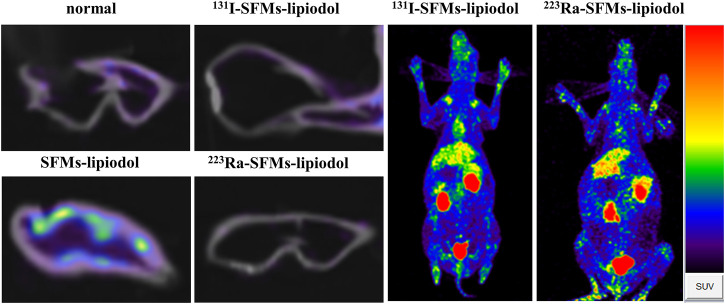
^68^Ga-DOTA-RGD PET/CT images of rabbit ears were captured at 4 days after embolization with ^131^I-SFMs-lipiodol.

To explore the influence of embolization on neovascularization, ^68^Ga-DOTA-RGD PET/CT was conducted at 4 days post-embolization. The uptake of ^68^Ga-DOTA-RGD was at the highest level in the SFMs-lipiodol group (SUV_max_ = 0.240 ± 0.040), confirming the inductive effect of embolization; the SUV_max_ of ^68^Ga-DOTA-RGD PET/CT in the normal group was 0.099 ± 0.036. In comparison, Figure 5 displayed that the tracer uptake increased in the ACA in the ^131^I-SFMs-lipiodol group (SUV_max_ = 0.141 ± 0.026), which indicated that new blood vessels or a new blood supply existed. In contrast, the uptake of ^68^Ga-DOTA-RGD in the ^223^Ra-SFMs-lipiodol group was at a low level (SUV_max_ = 0.053 ± 0.004), even lower than the normal group, manifesting a strong inhibition on neovascularization, which contributed to the accelerated necrosis shown in Figure 3. The results of ^68^Ga-DOTA-RGD PET/CT in each group were consistent with changes in the appearance of the rabbit ears.

### 3.5 The destruction of embolized vessels

#### 3.5.1 H&E staining

**FIGURE 6:**
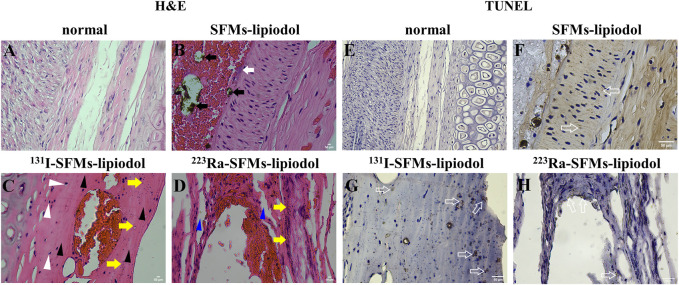
H&E staining of the longitudinal sections of the auricular central artery, normal **(A)**, 7 days after embolization with SFMs-lipiodol **(B)**, ^131^I-SFMs-lipiodol **(C)** and ^223^Ra-SFMs-lipiodol **(D)** (× 400). Black arrows indicate SFMs-lipiodol, white arrows indicate inflammatory cells, white triangular arrows indicate acidophilic bodies, yellow arrows cell indicate fragments, black triangular arrows indicate disappeared nuclei, blue triangular arrows indicate intercellular space; TUNEL staining of the longitudinal sections of the auricular central artery, normal **(E)**, after injection with SFMs-lipiodol **(F)**, ^131^I-SFMs-lipiodol **(G)** and ^223^Ra-SFMs-lipiodol **(H)** at 7 days post-embolization (× 400), hollow arrows indicate positive apoptotic cells.

The longitudinal sections of ACAs stained by H&E are displayed in Figure 6. In the normal group (Figure 6A), vascular endothelial cells and vascular smooth muscle cells were arranged in regular order. In Figure 6B, SFMs-lipiodol is indicated by black arrows in the lumen of the ACA at 7 days post-embolization. The SFMs-lipiodol group showed signs of moderate inflammation, indicating the post-treatment inflammatory cells, such as neutrophils and lymphocytes in the inside tissue of the vascular wall, which are indicated by a white arrow.

The organization structure of the vascular wall was changed in the H&E stained sections of the ^223^Ra-SFMs-lipiodol group (Figure 6D). In addition, nuclear atrophy and acidophilic bodies were found throughout the adjacent vascular tissues (white arrows). The majority of cell fragments were next to the lining of blood vessels (yellow arrows), and many nuclei disappeared in the lining of the ACA (black arrows). Compared with the ^131^I-SFMs-lipiodol group (Figure 6C), the rupture of the blood vessel wall was more distinct and thorough, and the normal vascular structures were difficult to discern in the ^223^Ra-SFMs-lipiodol group. The existence of intercellular space is indicated by blue arrows in Figure 6D, and some platelets entered the inner vessel wall, manifesting that Ra-223 could break the intercellular space.

#### 3.5.2 TUNEL staining

Only small amounts of cell apoptosis existed in normal tissues (Figure 6E). As exhibited in Figures 6F–H, cell apoptosis was detected in the inside of the ACAs in embolization groups by TUNEL staining (hollow arrows). In comparison with the normal group, the positive apoptotic cells were confined to the vascular endothelium in the ^223^Ra-SFMs-lipiodol group, while the involved range of cell apoptosis was wider in the ^131^I-SFMs-lipiodol group.

## 4 Discussion

This research aimed to establish the α-based TARE, which was first proved effective in inhibiting the proliferation of tumorous cells and VSMCs. Because the ability to proliferate independently of signals from other cell types is a fundamental characteristic of tumor cells (Kenny and Bissell, 2007), the direct break of vessels was meaningful in cutting off the blood supply of tumorous tissues. Thus, α-based TARE was an alternative to treating HCC, especially for a tumor close to the distal blood vessel.

Previous studies have confirmed that TARE is a safe technology with an objective response in phase 3 trials (Chauhan et al., 2018). The effect of TARE mainly relies on cutting the arterial supply and the radiation from radionuclides (Li et al., 2020). α particles have higher linear energy transfer than β particles (Lee et al., 2020). β particles require several hundred traversals through the nucleus to induce cell death, while less than ten α particles achieve the same efficacy (King et al., 2021). Compared to β particles, the high LET and short range mean that α radiation treatments are more suitable for small-volume or residual micro-tumors, potentially offering more efficient and specific killing of tumors and avoiding damage to surrounding normal cells. However, this shorter range limits the long-range “cross-fire” effect of α particles, which is advantageous in β particle therapies for large-volume tumors (Song et al., 2017). Hence, although there are great differences between the embolization influences of these two kinds of radionuclides, α particles have great potential as an alternative for personalized treatment, and the principle needs to be further explored.

This study preliminarily established a new direction of TARE on HCC treatment, titled α-based TARE. Although TARE was based on the hepatic artery embolization for unresectable HCC (Habib et al., 2015; Chen et al., 2022), the adjacent tissues of the hepatic artery need protection from scattered rays. In this consideration, Ra-223-based TARE, which has a short radiation range, could effectively kill the tumor and avoid radiation damage to the surrounding normal tissues. This research revealed that Ra-223-based TARE could lead to complete necrosis, and the properly confined radiation range minimized the occurrence of adverse effects. In addition, the short range of α particles makes it easier to carry out radiation protection even though the therapy nuclides have a relatively long half-life.

Most HCCs are highly vascularized. The upregulation of hypoxia-inducible factor proteins enhances the expression of proangiogenic factors, including vascular endothelial growth factor (VEGF), which promotes angiogenesis in HCC tumors (Morse et al., 2019). Consequently, inhibition of angiogenesis in tumors is expected to improve the effects of TARE treatment. The results of ^68^Ga-DOTA-RGD PET/CT indicated that new blood flow was gradually established to compensate for embolization in the ^131^I-SFMs-lipiodol group. When compared with the ^131^I-SFMs-lipiodol group, tracer uptake in the ^223^Ra-SFMs-lipiodol group was at a low level, even lower than the normal group (0.053 ± 0.004 vs. 0.099 ± 0.036, *p* < 0.01). These results showed that the Ra-223 radiation strongly inhibits angiogenesis, which is beneficial for patients with advanced HCC. ^223^Ra-SFMs-lipiodol could potentially inhibit the growth of a carcinoma and decrease the recurrence of the tumor. Hence, the development of α-based TARE is of great significance, and α-emitting radionuclides are promising candidates for TARE with their short range of radiation and high-energy α-particles.

The destruction of embolized vessels was another finding in this research. According to the analysis of H&E and TUNEL staining slides, the Ra-233 α radionuclides could break the intercellular space. Currently, resistance to targeted chemotherapeutic agents poses a huge challenge (Ma et al., 2021). This feature of breaking the intercellular space is far-reaching not only for cutting the artery feeding the tumor but also for the release of a drug to surrounding tissues. Thus, the use of chemotherapeutic drugs in TAE may decline when integrated with α-based TARE. For instance, the development of α-emitting radionuclides TAE together with chemotherapy drugs Sorafenib is a win-win option for the design of drugs for HCC treatment. Synergistic therapy could achieve the desired effect for HCC patients.

Several known challenges limit the wide application of α nuclides in the clinic (Marcu et al., 2018). For example, several low-dose radiation treatments over an extended period can improve effectiveness and reduce radiation toxicity. However, α-emitters are relatively scarce and are typically produced on a strict schedule, and it is critical that the supply of radionuclides is able to meet the clinical trial demand. In addition, although Ra-223 has the ability to be imaged *via* SPECT, the relatively low activity of the administered nuclides and the long acquisition time for SPECT produce blurred images. It is to be hoped that improving the quality and quantity of radionuclide production, combined with increasingly available tools, will make α nuclides suitable for clinical use in the future. In a word, α-based TARE has an application prospect for HCC therapy.

## 5 Conclusion

In this study, we primarily explored the effects of α-based TARE on the ACA as structural destruction of embolized vessels and inhibition of neovascularization. The embolization results of ^223^Ra-SFMs-lipiodol provided evidence for the therapeutic effects of α-based TARE and provided a reference for HCC treatments in the setting of a personalized TARE protocol.

## Data Availability

The original contributions presented in the study are included in the article/Supplementary Material; further inquiries can be directed to the corresponding authors.
